# Trichogranuloma in a Hairdresser with Systemic Sclerosis

**DOI:** 10.7759/cureus.2690

**Published:** 2018-05-25

**Authors:** Barbara Craveiro-Lopes, Ian Grant, Amanda I Adler

**Affiliations:** 1 General Medicine, Hospital Professor Doutor Fernando Da Fonseca; 2 Department of Plastic and Reconstructive Surgery, Addenbrooke’s Hospital, Cambridge University Hospitals Nhs Foundation Trust, Cambridge, Uk; 3 Wolfson Diabetes and Endocrine Clinic, Institute of Metabolic Science, Addenbrooke's Hospital, Cambridge University Hospitals Nhs Foundation Trust, Cambridge, Uk

**Keywords:** barber’s hand, scleroderma, occupational hand dermatosis, interdigital pilonidal sinus, prevention

## Abstract

Trichogranuloma is a rare occupational disease of hairdressers that develops when hair clippings penetrate the skin and cause a foreign-body reaction. We describe a case of a hairdresser with limited cutaneous systemic sclerosis who developed a chronic felon on her right third finger and received repeated courses of antibiotics without improvement. An occupational history and awareness of occupational diseases in hairdressers led to the correct diagnosis of trichogranuloma. Systemic sclerosis and concomitant finger ulcers may have predisposed the patient to this otherwise infrequent condition in an unusual location. Our case highlights the importance of occupational history and awareness of rare occupational diseases; we propose that treating and preventing skin disease may play a role in the prevention of trichogranuloma, thereby highlighting the need for protective measures in hairdressers.

## Introduction

Hairdressers and barbers have high rates of occupational skin diseases; in the United Kingdom, approximately 70% develop contact dermatitis [1]. Because of its prevalence, dermatologists diagnose contact dermatitis in hairdressers frequently; conversely, trichogranuloma (also known as barber’s hand, interdigital pilonidal sinus, or barber’s sinus [2]), is rarely reported, mostly seen by surgical specialties, and many clinicians are not aware of it [2-3]. Like a pilonidal sinus of the sacro-coccyx, it develops when cut or broken hairs penetrate the skin, causing a pyogranuloma. We report a case of a hairdresser with a history of systemic sclerosis that developed trichogranuloma, a constellation which has not been previously described.

## Case presentation

A 67-year-old right-handed female hairdresser working in a hair salon for 30 years with predominantly female customers presented to a plastic surgeon with a three-month history of a swollen, painful, and intermittently discharging lesion in the pulp of her third finger. She denied fever, chills, history of trauma, or having noted any foreign body in her wound. She reported a long-standing history of limited cutaneous systemic sclerosis, Raynaud’s syndrome, and frequent small ulcers at her fingertips. A swab grew coliform bacteria after she took repeated courses of antibiotics which failed to resolve her symptoms. The patient mentioned having to remove embedded hairs regularly from the skin of her hands, feet, and even her chest, but denied similar reactions in the past. Regular medication included ramipril and vitamin D.

**Figure 1:**
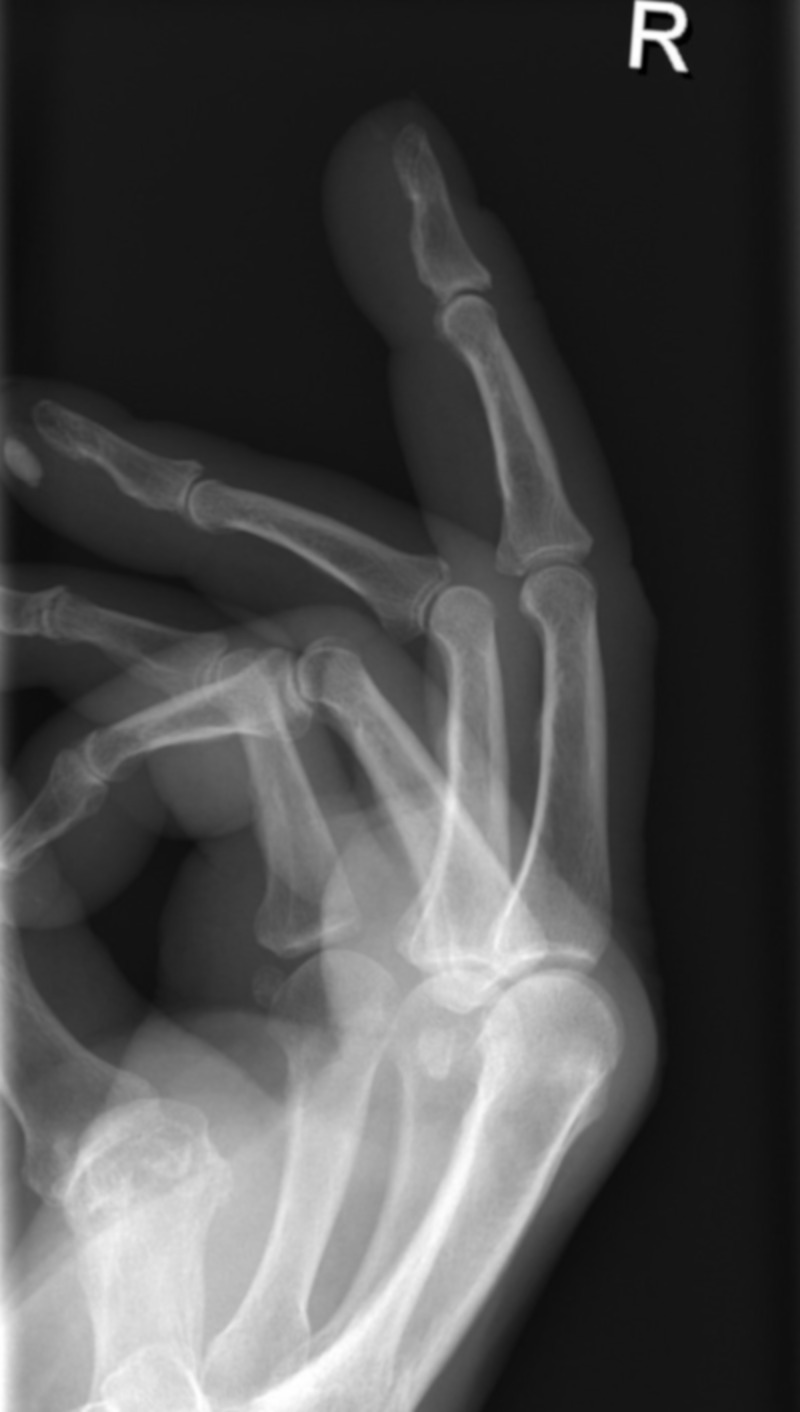
Lateral view of the right hand The affected digit reveals the presence of a possible foreign body. Note the calcinosis nodule on the pulp of the ring finger.

On physical examination, the surgeon documented a swollen, tender, erythematous, finger pulp with a small, crusty papule that did not produce discharge and was without evidence of lymphangitis. Plain radiographs suggested a foreign body, without osteomyelitis (Figure 1). Differential diagnoses included other foreign bodies from trauma; bacterial, viral or fungal infection, an arthropod bite, and calcinosis infection. A calcinosis nodule was noted in the pulp of her right fourth finger (Figure 1). Because of her occupation, the surgeon diagnosed pilonidal sinus with secondary abscess formation.

The surgeon recommended surgery and performed a fish mouth incision under local anaesthesia, incorporating an ellipse around the sinus, and excising the sinus cavity. The surgeon washed and sutured the wound. As the wound showed signs of infection three days post-operatively, the patient received a one-week course of oral amoxicillin/clavulanic acid. Sutures were removed on the twelfth post-operative day; however, because of renewed signs of infection, the patient took a further two-week course of oral flucloxacillin. The wound healed, and the patient returned to work one month after surgery. Three months postoperatively, she remains asymptomatic but has chosen not to use gloves.

## Discussion

Simple inquiries from the 1950s and 1970s have estimated a relatively high (7%-13%) prevalence of trichogranuloma in hairdressers [2,4]; it has been postulated that its frequently mild presentation rarely merits seeking medical attention [2], which could explain the relative scarcity in reported cases (around 20) [4] as well as lack of awareness among clinicians [2-3,5]. Histologically, trichogranuloma is a foreign-body granuloma around foreign implanted keratin; when cut hairs penetrate the epidermis, it proliferates reactively at the implantation site. At the dermo-epidermal level, a cyst-like mass develops which connects to the skin surface via epithelialized sinus tracts [2,6]. A nodule with a small (<1 mm) sinus opening develops, often with protruding hairs, swelling, erythema, and sero-purulent discharge [4]. Complications include abscess formation, lymphangitis, and osteomyelitis [6]. A marked male preponderance [2] has been explained historically, since male barbers traditionally have cut men’s hair [7] which, because of its characteristics, has been said to be more likely to penetrate skin [8]; this tendency may be changing, as nowadays females perform both hairdressing and barbering, comprising 87% and 69% respectively in the UK [1]. Trichogranuloma primarily affects hands, particularly the web spaces, since cut hairs tend to accumulate there [7] in the non-dominant, hair-holding hand [5]. However, as confirmed by our patient, hairdressers report that hairs constantly become embedded in various body parts, including in skin covered by underwear [9]. Most of these hair shaft injuries do not develop into symptomatic lesions, suggesting that specific factors may play a role in developing a symptomatic trichogranuloma. Proposed risk factors include prolonged wet work and contact with detergents, which soften the skin of the hands [2], as well as repeated cutting trauma [2,5], and cracks in calluses [9]. Onycholysis has been implicated in the development of subungual trichogranuloma [9-10]; it is plausible that other conditions, such as systemic sclerosis, may act similarly. Fingertip ulcers in systemic sclerosis patients are very common and result from recurrent ischemia and repeated microtrauma [11]. Our patient’s recurrent fingertip ulcers may have predisposed her to develop a trichogranuloma in an unusual location, as finger pulp trichogranuloma has been described only once previously [12].

There is no systematically-collected evidence on treating and preventing trichogranuloma. Preferred treatment generally consists of surgically excising the hair and inflamed tissue [3-4,8,13]; however, recurrence is possible [8], and may be influenced by wound closure as well as not employing further preventative measures [4-6,8]. In accordance with current evidence on sacrococcygeal pilonidal sinus treatment [14], most authors agree that secondary wound healing leads to lower recurrence, but also leads to prolonged healing and worse scarring [3-4]. Because of our patient’s scleroderma and its association with delayed wound healing [11], primary closure was chosen. So far, suggested preventative measures include using open-ended gloves (to retain pulp sensitivity) while cutting [5-6,10], barrier creams [4], and adhesive strips [8], as well as washing and drying hands between clients and removing embedded hairs as soon as possible [6,8]. There is no evidence for any of these options; furthermore, some of them may not be helpful, as fingerless gloves would not have prevented our case. Assuming trichogranuloma is more likely to develop in at-risk skin, encouraging evidence-based health and safety recommendations that prevent skin damage and disease in hairdressers, as recommended by the UK Health and Safety Executive [1], seems reasonable. Wet work and contact with chemicals have been identified as major contributors to skin disease and laceration in hairdressers [1]; wearing gloves during wet-work (ideally single use, powder and latex-free and properly fitted [1]) should be encouraged. It follows that for patients with pre-existing skin conditions, preventive measures are even more important. Also, it seems logical to treat any underlying skin lesions; that is, to treat the primary condition rather than attributing trichogranuloma to occupation alone [9]. Our case supports this notion, as better control of ulcers could arguably have prevented the development of this condition.

## Conclusions

We present the case of a hairdresser, with a history of limited cutaneous systemic sclerosis, who developed trichogranuloma in the pulp of her right third finger. We highlight the importance of occupational history; propose that individual risk factors, such as a history of chronic ulcers, may play a role in the pathophysiology of symptomatic trichogranuloma; and lastly, we suggest that preventing occupational trichogranuloma may include controlling underlying disease and adhering to skin protection guidelines.

## References

[1] (2018). Health and safety executive worksheet: occupational skin disease in hairdressers and barbers. Occup. Ski. Dis. Hairdressers Barbers.

[2] Stenveld H (2012). Hairdressers. Kanerva’s Occupational Dermatology.

[3] Eryilmaz R, Okan I, Ozkan O V, Somay A, Ensari CO, Sahin M (2012). Interdigital pilonidal sinus: a case report and literature review. Dermatologic Surg.

[4] Gul V, Destek S, Etkin E (2016). Approach to inter digital pilonidal sinus: our clinical experience and literature review. Int J Surg Res Pract.

[5] Stahl S, Ben-Asher Y (2000). Foreign body hair granuloma in barbers. Eur J Plast Surg.

[6] Schröder CM, Merk HF, Frank J (2006). Barber’s hair sinus in a female hairdresser: uncommon manifestation of an occupational dermatosis. J Eur Acad Dermatology Venereol.

[7] Patel MR, Bassili L, Nashad R, Anselmo MT (1990). Barber’s interdigital pilonidal sinus of the hand: a foreign body hair granuloma. J Hand Surg Am.

[8] Adams C, Petrie P, Hooper G (2001). Interdigital pilonidal sinus in the hand. J Hand Surg Br.

[9] de Berker DAR, Dawber R, Wojnarowska F (1994). Subungual hair implantation in hairdressers. Br J Dermatol.

[10] Nagtzaam IF, van der Velden JJAJ, Kelleners-Smeets NWJ, Frank J (2007). Onycholysis associated with subungual manifestation of barber’s hair sinus. Int J Dermatol.

[11] Fox P, Chung L, Chang J (2013). Management of the hand in systemic sclerosis. J Hand Surg Am.

[12] Grant I, Mahaffey PJ (2001). Pilonidal sinus of the finger pulp. J Hand Surg Am.

[13] Stern PJ, Goldfarb CA (2004). Interdigital pilonidal sinus. N Engl J Med.

[14] (2018). Pilonidal sinus disease - NICE CKS. https://cks.nice.org.uk/pilonidal-sinus-disease.

